# Current management and prognostic features for gastrointestinal stromal tumor (GIST)

**DOI:** 10.1186/2162-3619-1-14

**Published:** 2012-06-18

**Authors:** Gurpreet Lamba, Ridhi Gupta, Byung Lee, Samir Ambrale, Delong Liu

**Affiliations:** 1 Division of Oncology/Hematology, New York Medical College and Westchester Medical Center, Valhalla, NY, 10595, USA

## Abstract

Stromal or mesenchymal neoplasms affecting the gastrointestinal (GI) tract have undergone a remarkable evolution in how they are perceived, classified, approached, diagnosed and managed over the last 30 years. Gastrointestinal stromal tumors (GIST) account for approximately 1% to 3% of all malignant GI tumors. The clinical features can vary depending on the anatomic location, size and aggressiveness of the tumor. Metastatic GIST represents a successful example of molecular targeted therapy. In this comprehensive review, we discuss the epidemiology, clinical features and diagnostic modalities for GIST. We also describe treatment options for early stage, locally advanced and metastatic GIST. Indications for neoadjuvant and adjuvant therapy along with duration of therapy are also explained. A brief discussion of latest biomarkers and updates from recent meetings is also provided.

## Introduction

**Figure 1:**
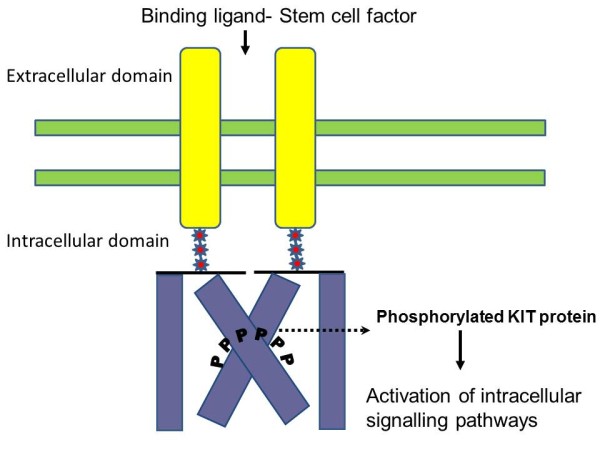
**The structure of KIT/CD117 and the signal transduction.** KIT is a transmembrane receptor type tyrosine knase. The stem cell factor/KIT ligand binds to KIT and activates the KIT tyrosine kinase. The phosphorylated (activated) KIT then activates its substrates which lead to cell proliferation.

Stromal or mesenchymal neoplasms affecting the GI tract have undergone a remarkable evolution in how they are perceived, classified, approached, diagnosed and managed over the last 30 years. A major breakthrough occurred with the discovery of expression of the CD117 antigen by almost all gastrointestinal stromal tumors (GIST)[1]. The other group of spindle cell neoplasms arising in the GI tract includes lipomas, schwannomas, hemangiomas, usual leiomyomas and leiomyosarcomas are typically CD117-negative [2]. The CD117 molecule is part of the KIT (c-kit) receptor tyrosine kinase that is a product of the KIT proto-oncogene (Figure 1). GIST research and clinical care sets another great example of translational research that turns laboratory discovery to successful clinical application. From this fundamental mechanistic understanding of GIST, a series of worldwide investigations and trials have developed novel and effective ways to approach patients with this disease. In this review, we discuss the basics of GIST and highlight recent advances and their relevance to current clinical practice as well as future directions.

### Epidemiology

GISTs account for approximately 1% to 3% of all malignant GI tumors [3]. Epidemiologic data such as that from Surveillance, Epidemiology and End Results (SEER) program are difficult to interpret since the earlier definition of "malignant GIST" was derived from criteria published in 1990, before GIST was molecularly characterized [4]. The estimated incidence of GIST has been revised upward to approximately 5,000 new cases per year in the United States (US) [5,6]. The most dependable international epidemiologic data are from population-based studies that reexamined all cases of potential GIST [7-10]. These studies reported annual incidence of GIST ranging from 11 to 14.5 per million population. More recent studies suggest that the incidentally detected sub-centimeter gastric GIST lesions may be more frequent than expected [11,12].

### Clinical presentation

The clinical features can vary depending on the anatomic location, size and aggressiveness of the tumor. Most symptomatic patients have tumors larger than 5 cm in maximal dimension. In a series of cases with leiomyomas and leiomyosarcoma (without separation of the GISTs), there were three major presentations [13], GI bleeding (40%), abdominal mass (40%) and abdominal pain (20%). Two-thirds of patients had GI bleeding while 25 to 40% presented with an intestinal obstruction. Intestinal perforation can also occur uncommonly. Rare patients have been described who present with severe hypoglycemia due to para-neoplastic production of insulin like growth factor II [14]. Other symptoms at presentation may include nausea, vomiting, anorexia, and weight loss. The vast majority of GIST metastases at presentation are intra-abdominal, either to the liver, omentum or peritoneal cavity [15]. Metastases to lymph nodes or to extra-abdominal sites via lymphatics are rare.

### Diagnostic imaging

GIST should always be among the differential diagnosis of an intra-abdominal non-epithelial malignancy. CT is essential for evaluating the primary tumor and for accurate staging. Magnetic resonance imaging (MRI) has a comparable diagnostic yield [16] and lacks radiation exposure. However, CT is preferred initially for screening and staging. CT is better at global evaluation of the abdomen, especially the hollow viscera, than MRI. MRI may be preferred for GISTs at specific sites, such as rectum or liver. Tumors that are greater than 5 cm, lobulated, enhance heterogeneously, and have mesenteric fat infiltration, ulceration, regional lymphadenopathy, or an exophytic growth pattern on CT are more likely to metastasize [17-21]. In contrast, GISTs with less metastatic potential tend to enhance in a homogeneous pattern, and often show an endoluminal growth pattern. CT or MRI scanning can assess the decrease in lesion density which can be an early marker of beneficial response in GIST patients treated with TKI drugs [22]. Routine clinical practice rarely requires Positron Emission Tomography (PET) imaging of GIST for clinical care [23]. PET imaging may have the advantage of detecting small lesions at least 1 cm in size because neither the normal bowel nor omentum takes up the fluorodeoxyglucose (FDG) tracer with excess avidity. The reported sensitivity of PET for GIST is 86 to 100% [24,25]. PET can be useful for detecting an unknown primary site or resolving ambiguities from CT [26]. On upper GI endoscopy, a smooth, mucosa-lined protrusion of the bowel wall, with or without signs of bleeding and ulceration may be seen [27]. However, endoscopic biopsies using standard techniques usually do not obtain sufficient tissue for a definite diagnosis [28]. Endoscopic ultrasound (EUS) -guided fine-needle biopsy forceps also may not yield enough tissue, but might exclude other lesions that arise sub-mucosally. Snare biopsies can result in perforation and generally should be avoided except in carefully selected cases [28]. A preoperative biopsy is not generally recommended for a resectable lesion in which there is a high suspicion for GIST, and the patient is otherwise operable. However, a biopsy should be done to confirm the diagnosis particularly when metastatic disease is present or suspected. If preoperative imatinib is considered in a patient who has a large locally advanced lesion thought to represent GIST, a biopsy should be done. An EUS-guided biopsy (in carefully selected patients and preferably of the primary lesions) is more desirable than a percutaneous biopsy [29].

### Histopathology and cytology

Differentiation between GISTs and other tumors is typically based upon immunohistochemistry (IHC) and molecular analysis. Histologic findings seen on hematoxylin and eosin-stained sections do not reliably or specifically relate to the immunophenotype nor the molecular genetics of the lesions [30]. GISTs characteristically stain positive for CD117 antigen (KIT) on IHC assays. The level of expression can vary from generally diffuse and strong (most common in the spindle cell subtype) to focal and weakly positive in a dot-like pattern (the epithelioid subtype) [5]. CD34 expression is not specific for GIST and it can also be seen in desmoid tumors [5,31,32]. About 95% of GISTs are KIT-positive, while 60 to 70% are positive for CD34, 30 to 40% are positive for smooth muscle actin, 5% for S-100 protein, and 1 to 2% are positive for desmin or keratin [2,5,32]. Identification of CD117-negative GIST remains a diagnostic challenge, and these are most likely to be driven by alternative kinases like PDGFRA [33]. The antigen known as DOG-1 (“discovered on GIST-1”) can also help to identify certain KIT-negative GIST lesions as DOG-1 expression is quite specific for GIST [34,35].

### Prognostic features of GIST

The current consensus is to treat all GISTs, including those with a benign appearance by conventional histopathologic criteria, as having the potential to behave in a malignant fashion [5,23]. This is due to the biologic behavior of GIST, which can be highly variable and with long follow-up, virtually all GISTs have the potential for malignant behavior, even those 2 cm or less with bland histologic features [2]. Thus, it is not appropriate to define any GIST as "benign" per se [5,36,37]. The most reliable prognostic factors for GIST are size of the primary tumor and the mitotic index. Additionally, recurrence and survival rates can be affected by the location of the primary GIST lesion (e.g. with small bowel and rectal primary GIST demonstrate worse prognosis than gastric GISTs) [36,37]. PDGFRA mutations (almost always in gastric primaries) appear to be a very favorable prognostic factor for low risk of recurrence [38]. Among patients with GIST, histologic type may also impact prognosis. In a report of 48 patients, the five-year recurrence-free survival rate was significantly higher among patients with spindle cell as compared to epithelioid or mixed histology [39]. However, others report a prognostic influence of the degree of cellularity but not histologic subtype [40,41].

### Management paradigms for early-stage GIST

The natural history of early-stage GIST has been documented in single-institution studies. Two hundred patients with GIST were followed prospectively at the Memorial Sloan-Kettering Cancer Center [15]. Eighty of these patients who had primary disease were managed with complete surgical resection. This group of patients demonstrated a 5-year disease-specific survival rate of 54%. On multivariate analysis, large tumor size (>10 cm) was the only negative predictive factor on disease-specific survival. Definitive surgery remains the treatment of choice for patients with localized GIST. Complete surgical resection is recommended for small gastric GISTs <2 cm at high risk of recurrence based upon EUS appearance (irregular borders, cystic spaces, ulceration, echogenic foci, or heterogeneity in appearance). For tumors that lack these features, endoscopic surveillance is an option. However, initial therapy with imatinib may be preferred if a tumor is borderline resectable, or if resection would necessitate extensive organ disruption. There is no specific consensus or guideline for the use of neoadjuvant imatinib at this time. However, this could serve as the initial therapeutic intervention, with follow-up at close intervals to ensure appropriate response to therapy. In these situations, early assessment of therapeutic response by ^18^FDG-PET scanning could be very valuable to confirm the response to imatinib. After maximal response (usually occurring within 4 to 6 months), definitive surgery could be performed [42].

### Adjuvant therapy for resected early-stage GIST

A large multicenter landmark phase 3 trial (ACOSOG study Z9001) appears to support the use of imatinib therapy in patients with larger, fully resected, primary localized GIST lesions at significant risk of relapse [43]. Seven hundred and thirteen adults with a completely resected primary GIST (> = 3 cm, KIT+) were randomly assigned to one year of adjuvant imatinib (400 mg daily) or placebo [43]. The trial was stopped at an early, preplanned, event-based interim analysis because of the positive outcome. At a median follow-up of 20 months, 30 patients in the imatinib group recurred or died, versus 70 in the placebo group (8 versus 20%). The one-year relapse-free survival (RFS) rate was 98 versus 83% favoring imatinib, with a hazard ratio for RFS of 0.35 (95% CI 0.22 to 0.53). In a later analysis, the benefit was greatest in those with high-risk disease (relapse rate 47 versus 19% for placebo and imatinib, respectively); for moderate risk disease it was 14 versus 5%, respectively [44]. Despite prolonged RFS, no benefit in overall survival (OS) was noted with short follow-up. Furthermore, after the study was unblinded, all patients randomized to placebo were allowed to crossover to active treatment, thus obscuring any potential differences in overall survival between the groups. Thus, it remains unclear whether imatinib is simply delaying or really preventing relapses. The trial tested only the 400 mg daily dose. Patients with advanced GIST and KIT exon 9 mutations do better with 800 mg daily doses. Whether doses greater than 400 mg should be used in the adjuvant setting will require prospective study. Although they were excluded, these results may also be significant for the 4% of patients with GISTs that lack KIT overexpression, but have mutations in KIT or PDGFRA, and can respond to imatinib [45]. Contrary to this, patients who lack detectable KIT or PDGFRA mutations or who have specific mutations that are known to be resistant to imatinib may not benefit. Whether such patients should be identified prospectively and specifically excluded from receiving adjuvant imatinib is unclear.

The FDA approved the use of imatinib as adjuvant therapy without any qualification or restriction following resection of primary GIST ≥3 cm in size, but the European Medicines Agency approved adjuvant imatinib only for the subset of GIST patients with primary disease that is judged to be “at significant risk of recurrence” following resection.

#### *Estimation of recurrence risk*

Several criteria have been proposed, originally to classify the malignant potential of a GIST. Tumor size, mitotic rate, and site of tumor origin have gained the greatest acceptance as being predictive of outcome [46]. Risk stratification models have also been proposed to distinguish prognosis in resected GIST [39]. Tumors arising from the small bowel, colon, rectum, or mesentery are associated with less favorable outcomes than those arising from the stomach [37]. Thus, each case must be approached individually, balancing the estimated probability of a disease recurrence.

Adjuvant imatinib for 1 year certainly has a major impact on disease control rates, but these differences appear to fade with increased rates of recurrence noted on discontinuation of the imatinib dosing. It is possible that a longer duration of adjuvant therapy might further improve clinical outcomes. A small study of adjuvant therapy for 2 years in Seoul, Korea has shown much higher rates of disease control than in the 1-year study, consistent with biological expectations [47]. The results of a trial by the Scandinavian Sarcoma Group (SSG) XVIII trial were presented at the 2011 American Society of Clinical Oncology (ASCO) meeting and then published in 2012. This trial compared 36 versus 12 months of imatinib (400 mg daily) in 400 patients with high-risk resected GIST [48]. High-risk was defined as having at least one of: tumor size >10 cm, mitotic count >10/50 high-power fields (hpf), tumor size >5 cm with mitotic rate >5/50 hpf, or tumor rupture. About one-half of the enrolled patients had gastric primary tumors. At a median follow-up of 54 months, prolonged treatment was associated with a significant improvement in RFS, the primary endpoint (five-year RFS 66 versus 48%, HR 0.46, 95% CI 0.32 to 0.65) as well as OS (92 versus 82%, HR 0.45, 85% CI 0.22 to 0.89). However, twice as many patients discontinued imatinib for reasons other than disease progression in the prolonged therapy group (26 versus 13%). This dataset attempts to establish 36 months of adjuvant imatinib as a new standard for patients with high-risk resected GIST. In both groups, within 6 to 12 months of discontinuing adjuvant imatinib, rates of disease recurrence were similarly increased. This and previous findings seem to support the notion that recurrences are just delayed, rather than being prevented. The question now is whether treatment should be continued for longer than three years. One study assessed cost-effectiveness of 3 years versus 1 year of adjuvant imatinib in the US from a payer’s perspective [49]. They reported total lifetime cost per patient at $302,100 with 3 years versus $217,800 for 1 year of imatinib therapy. They also found that patients on 3 years of imatinib had higher quality-adjusted life years (QALYs). Thus, incremental cost effectiveness ratio of 3 years versus 1 year of imatinib therapy was $62,600/QALY. At a threshold of $100,000/QALY, 3 year imatinib therapy was cost-effective. At the same meeting Deflin and colleagues reported on the comparison of the clinical benefit of an adjuvant therapy in GIST with other adjuvant cancer therapies [50]. They showed that imatinib has one of the lowest number needed to treat amongst other adjuvant treatments, at 1 and 3 year of follow-up. Thus, both clinical and economic results now suggest treating surgically resected GIST patients with 3 years of imatinib would result in improved quality-adjusted and OS.

The Intergroup EORTC 62024 trial with randomization between two years of imatinib and observation alone has been completed and is awaiting data maturation. OS is the primary end point. A single-arm phase II five-year adjuvant imatinib trial, PERSIST5, has also completed accrual; data probably will not be available for several years.

Additionally, it is also important to assess whether certain GIST genotypic subsets benefit more—or fail to benefit at all—from adjuvant therapy with TKIs. The ACOSOG Z9001 randomized trial has confirmed certain differences between the behaviors of genetically different forms of GIST. The KIT exon 11 deletion confers a much higher risk of relapse compared with other mutational subtypes in the placebo arm, but these exon 11 mutants benefit from adjuvant imatinib to ablate this added risk [38]. The PDGFRA-driven GISTs are often more aggressive in the metastatic setting, however, they are remarkably indolent with a low risk for relapse following resection of limited-stage primary disease, similar to the wild type GIST. Given the potential toxicities and costs of imatinib, as well as the tremendous success of imatinib for recurrent disease, it is very important that adjuvant studies of imatinib be completed and analyzed in the context of molecular subtyping.

### Neoadjuvant therapy

There are no published randomized trials addressing the benefit of neoadjuvant imatinib in the management of GIST. Since 2003, several case reports and small retrospective series have been published. These include a mix of patients with borderline resectable and unresectable primary disease, as well as metastatic and locally recurrent disease that is potentially amenable to gross resection [51-57].

The multicenter RTOG (Radiation Therapy Oncology Group) 0132/ACRIN (American College of Radiology Imaging Network) 6665 trial assessed neoadjuvant imatinib either in primary resectable GIST or as a planned preoperative cytoreduction agent for metastatic GIST [58]. Patients with primary GIST (≥5 cm, Group A) or operable metastatic/recurrent GIST (≥2 cm, Group B) were treated with neoadjuvant imatinib (600 mg/day) for approximately two months and maintenance imatinib after surgery for 2 years. The clinical outcomes including progression-free survival (PFS), disease-specific survival (DSS), and overall survival (OS) at a median follow-up of 5.1 years were presented at ASCO 2011 meeting [59]. The authors correlated these endpoints with duration of imatinib therapy. Sixty-three patients were originally entered (53 analyzable). There were 31 patients in Group A and 22 in Group B. Estimated 5-year PFS and DSS were 57% A, 30% B; and 77% A, 77% B, respectively. Estimated 5-year OS was 77% for A, and 68% for B. Median time to progression had not been reached for Group A and was 4.4 years for Group B. In Group A, 7/11 patients progressed > 2 years from registration; 6/7 progressing patients had stopped imatinib prior to progression. In Group B, 10/13 patients progressed >2 years from registration; 6/10 progressing patients had stopped imatinib prior to progression. This long-term analysis suggested a high percentage of patients progressed after discontinuation of 2-year maintenance imatinib therapy following surgical resection. This trial confirmed the safety of neoadjuvant imatinib. The available data from patients treated for advanced disease suggest that maximal radiographic response to imatinib generally occurs within three to nine months.

Data from retrospective series also support the benefit of initial imatinib therapy [52-56,60]. The largest experience consisted of 46 patients who underwent surgery after imatinib. Eleven patients had a locally advanced primary tumor, while 35 had recurrent or metastatic disease [52]. All 11 patients who were treated for a locally advanced primary tumor (median 12 months of imatinib prior to resection had one complete and eight partial responses as assessed by CT) successfully underwent complete surgical resection. There was one complete pathologic response. At a median follow-up of 19.7 months post-resection, all 11 were alive and ten of them were recurrence-free. There were 11 partial responses among the 35 patients treated for recurrent/metastatic disease. Eleven patients were able to undergo a compete resection including two with a complete pathologic response. Patients with an objective partial response to imatinib (by CT) were significantly more likely to undergo a complete resection. All eleven of the completely resected patients were alive at a median follow-up of 30.7 months, but six recurred at a median of 15 months, despite continuation of imatinib therapy.

A small trial suggested response rates of up to 70% after very brief periods of neoadjuvant imatinib (three to seven days), as assessed by FDG-PET and dynamic CT [61]. However, in this small prospective randomized phase II trial, there was no evidence of histologic cytoreduction (and therefore, no potential benefit in terms of reduced tumor bulk) from ≤ 7 days of neoadjuvant imatinib, and no suggestion that intra-operative blood loss was reduced, even though blood flow to the tumor was reduced as measured by dynamic CT. Thus, the clinical benefit of very short periods of neoadjuvant imatinib (termed "nano-neoadjuvant therapy") [62] is unproven. It did prove that radiographic responses and tumor cell apoptosis occur within the first week of imatinib therapy.

Guidelines from the NCCN recommend initial treatment with imatinib for patients with marginally resectable tumors and for those who have potentially resectable disease but with the risk of significant morbidity. A daily dose of 400 mg per day is the usual approach, although if a KIT exon 9 mutation is identified, dose escalation to 800 mg per day is reasonable. This is also recommended in ESMO guidelines [63].

### Management of metastatic, unresectable or recurrent GIST

Various mechanisms are responsible for the resistance of GISTs to conventional cytotoxic chemotherapy. In one study evaluating the differences in outcome between GIST and leiomyosarcomas, significantly higher levels of expression of P-glycoprotein (38.4% vs. 13.4%) and MRP1 (35.4% vs. 13.3%) were demonstrated in the GIST cells [64].

During the 1990’s, screening studies [65-67] demonstrated that a signal transduction inhibitor 571 (STI 571, imatinib mesylate) inhibits the tyrosine kinase activity of BCR-ABL as well as KIT. A multicenter US–Finland collaborative study enrolled 147 patients with metastatic GIST between July 2000 and April 2001 [68]. Radiographic responses were seen within six months in 54% patients. These results along with the outcomes of the European Organization for Research and Treatment of Cancer (EORTC) trial 75 [69] confirmed the unparalleled activity of imatinib in controlling metastatic GIST. Despite these excellent results complete responses are rare (less than 10%), and most patients who initially respond ultimately acquire resistance via additional mutations in KIT. The median time to progression is roughly two to three years [68,70-72], although it is longer in other series [73].

Correlative science studies have reported that the type of mutation in KIT and PDGFRA correlates with clinical response [74-77]. High dose imatinib may preferentially benefit patients with exon 9 mutations [77,78]. However, no differences in OS between low-dose and high-dose imatinib in patients with exon 9 mutations was seen [79].

Rapid disease progression was seen within months after the imatinib is stopped [80,81]. At ASCO 2011, BFR 14 trial reported the effect of interruption of imatinib therapy in patients with GIST [82]. At the same meeting, Domont et al. reported the influence of imatinib interruption and reintroduction on tumor burden in patients with GIST on the BFR 14 trial. Among randomized patients with imatinib interruption 49% experienced progressive disease of the known tumor while 51% had new lesions with concomitant progression of known lesions [83]. Thus, continuous therapy until disease progression (or lifelong if disease does not progress) is currently standard of care.

Imatinib can rapidly and dramatically decrease tumor avidity for ^18^FDG [84-86]. With PET imaging, the down-modulation of tumor avidity for FDG is far earlier than changes noticeable on CT scanning [68,84,86]. Thus PET scans can aid in the detection of primary and secondary imatinib resistance. CT criteria using either no growth in tumor size or a combination of tumor density and size criteria have shown a close correlation with the predictive value results of FDG-PET [22,87]. Imatinib therapy can also change the density of tumor masses in GIST [22,68,88]. This is an important early clinical marker of antitumor activity [89,90].

### Resistance to imatinib

Clonal evolution of resistant GIST may be detected after a durable objective response and disease control. Several mechanisms of resistance to imatinib in GIST may exist [91,92]. Pharmacokinetic variability may also contribute to drug resistance [93]. Limited clonal progression appears as the first sign of resistance to imatinib [55,92,94]. Dose escalation from imatinib 400 mg daily may be considered for those with clear evidence of disease progression [45,95]. Sunitinib targets multiple tyrosine kinases, including the vascular endothelial growth factor receptors and PDGFR. An increasing number of reports indicate efficacy for the multi-targeted TKI sunitinib in imatinib-refractory or intolerant patients [95-98]. Sunitinib has become the current standard of care for patients who have failed imatinib.

As with imatinib, the clinical activity of sunitinib is significantly influenced by the specific mutation type. Resistance to sunitinib shares similar pathogenetic mechanisms to those identified in imatinib failure, with acquisition of secondary mutations after an extended initial response to the drug [99].

Sorafenib and other TKIs (i.e. sorafenib, dasatinib, motesanib, nilotinib) have been studied in refractory GIST or after resistance to imatinib and/or sunitinib [1,100-105]. The efficacy of sorafenib was addressed in a multicenter phase II trial involving patients with either imatinib or imatinib and sunitinib-refractory GIST [106]. In a preliminary report presented at the 2011 ASCO GI Cancers symposium, the disease control rate (defined as the proportion of patients without progression as the best radiologic response) was 68%, and median PFS was 5.2 months.

Nilotinib was studied in a randomized phase 3 clinical trial (ENEST g3) [107]. In this trial nilotinib was compared to a heterogeneous control arm in patients advanced/metastatic GIST who had failed imatinib and sunitinib. The control arm included best supportive care with physician choice to continue or stop imatinib or sunitinib. It failed to show significant benefit for nilotinib. Some of the most promising new approaches to overcome resistance to TKIs in GIST include targeting multiple levels of the signal transduction cascade by combining agents. This has been done, for example, by combining a kinase inhibitor such as imatinib with an inhibitor of the mTOR downstream signaling partner using the mTOR inhibitor everolimus [108]. Other strategies that are being explored include the inhibition of other pathways critical to the molecular processing of the mutant KIT or PDGFRA oncoproteins, such as the chaperone function of the heat shock protein-90 system. By inhibiting heat shock protein-90, preclinical and early clinical studies have already documented antineoplastic effects on kinase-inhibitor-resistant GIST both in vitro and in patients with progressive disease [109,110].

### New prognostic features

The survival was higher in GIST patients who had PDFGRA mutation as compared to KIT mutations. In patients with KIT mutations, point mutations and duplication in KIT axon 11 had better survival than GIST with other KIT mutations [111].

In a single center study of GIST patients who underwent curative surgery and were not treated with imatinib, the 2 year RFS was lower in patients who had both 557 and 558 codon mutations than in those with either 557 or 558 mutated (*p* = 0.03) [112].

Hypertension (HTN) is a side effect of drugs with VEGF signaling pathway inhibition. The relationship between sunitinib- associated HTN and treatment efficacy was analyzed. The results significantly favored the patients who developed HTN while on treatment with Sunitinib. These patients had significantly prolonged OS, PFS and TTP as compared to patients who did not develop HTN. Development of HTN during sunitinib therapy may therefore be used as a biomarker for anti-tumor efficacy [113].

At ASCO GI 2012, a mitotic index of 5/50 HPF was reported to be equivalent to a standardized uptake value (SUV) of 4.3, and a Ki67 labeling index of 5 was equivalent to SUV of 6.3 on a PET-CT. Thus, an SUV of 5 can predict the malignant potential between the high and low/intermediate risk [114].

D-dimer may be another potential marker in GIST [115]. Radiological progression (rPD) was associated with higher d-dimer levels. D-dimer levels <1000 had a negative predictive value for rPD of 85%. Thus, d-dimer test may reduce the burden of CT scanning in a useful percentage of patients but will require further validation.

### Recent trends

Billimoria et al. reported the evolution of multimodality management of GIST with adjuvant and neoadjuvant therapy. They found that between 2001 and 2007 use of adjuvant therapy with imatinib increased from 27% to 47%. Use of neo-adjuvant therapy increased from 0% to 15% in patients with tumors >6 cm [116]. Although the incidence of GIST increased from 1998 to 2001, it remained stable from 2001 to 2007. This period also saw a significant decrease in patients referred for surgery. Survival of patients diagnosed in 2005–2007 group improved over those diagnosed from 2002–2004 for both resected and unresected tumor, with resection of the tumor being an independent predictor of survival [117].

Italiano and co-workers reported the pattern of care, prognosis and survival in patients treated with first line Imatinib or second line Sunitinib in patients with GIST over 9 centers, including 176 patients. In the preliminary results they found that patients having secondary mutations and low serum albumin levels had the worst outcome [118].

#### *Updates from the BFR 14 trial*

BFR 14 is a prospective multicenter study from 2002 to 2009 which enrolled 434 patients. Blesius et al. reviewed 236 patients who were started on imatinib 400 mg daily and had been on it for 5 years. Patients who did not show any progression were retrospectively analyzed. They found that patients with small tumor volume at inclusion, good performance status, having exon 11 mutation in vicinity of codon 557–558 have higher sensitivity to imatinib and have a prolonged outcome as compared to other patients [119]. Bertucci and associates investigated factors predicting long term prognosis in patients with advanced GIST on the BFR 14 trial. The study found that female sex, performance status of 0, platelet count <400,000/dl, lymphocyte count >1500/mm^3^ were independent predictors of overall survival. Patients with CD 34 positivity on tumors have a better PFS [120].

## Conclusions and future directions

With the molecular signature of CD117/KIT mutation, GIST has provided a great model for targeted therapy. Novel targeted agents are being explored [1]. Combination therapy of TKI inhibitors either concurrently or sequentially with agents of different classes may have synergistic effects. It is therefore predictable that further clinical research by combining agents with novel mechanisms of action for this challenging malignancy will be forthcoming [121-124].

## Competing interests

The authors have no conflicts of interests.

## Authors’ contributions

SA, RG, BL contributed to data preparation. GL and DL were involved in concept design, data collection, and manuscript preparation. All authors reviewed and assisted in revising the manuscript. All authors read and approved the final manuscript.
